# Effect of Hemoadsorption for Cytokine Removal in Pneumococcal and Meningococcal Sepsis

**DOI:** 10.1155/2018/1205613

**Published:** 2018-06-19

**Authors:** Francesca Leonardis, Viviana De Angelis, Francesca Frisardi, Chiara Pietrafitta, Ivano Riva, Tino Martino Valetti, Valentina Broletti, Gianmariano Marchesi, Lorenza Menato, Roberto Nani, Franco Marson, Mirca Fabbris, Luca Cabrini, Sergio Colombo, Alberto Zangrillo, Carlo Coniglio, Giovanni Gordini, Lucia Stalteri, Giovanni Giuliani, Vittorio Dalmastri, Gaetano La Manna

**Affiliations:** ^1^Intensive Care Unit, Fondazione Policlinico Tor Vergata, Viale Oxford 81, 00133 Roma RM, Italy; ^2^Intensive Care Unit 3, Department of Anesthesia and Intensive Care, ASST Papa Giovanni XXIII, Piazza OMS 1, 24127 Bergamo BG, Italy; ^3^Intensive Care Unit, Azienda ULSS 2 Marca Trevigiana, Presidio Ospedaliero di Treviso, Piazzale dell'Ospedale 1, 31100 Treviso TV, Italy; ^4^Anesthesia and Intensive Care Unit, IRCSS San Raffaele, Via Olgettina 60, 20132 Milano MI, Italy; ^5^Resuscitation and Territorial Emergency Unit, Maggiore Hospital, Largo Nigrisoli 2, 40133 Bologna BO, Italy; ^6^Nephrology, Dialysis and Transplantation Unit, Azienda Ospedaliera-Universitaria di Bologna, Via Massarenti 9, 40138 Bologna BO, Italy

## Abstract

Bacterial meningitis and septicemia are invasive bacterial diseases, representing a significant cause of morbidity and mortality worldwide. Both conditions are characterized by an impressive inflammatory response, resulting rapidly in cerebral edema, infarction, hydrocephalus, and septic shock with multiple organ failure. Despite advances in critical care, outcome and prognosis remain critical. Available adjunctive treatments to control the inflammatory response have shown encouraging results in the evolution of patients with sepsis and systemic inflammation, but meningococcal or pneumococcal infection has not been investigated. We herein report five patients with similar critical pathological conditions, characterized by pneumococcal or meningococcal sepsis and treated with hemoadsorption for cytokine removal. All patients showed a progressive stabilization in hemodynamics along with a rapid and marked reduction of catecholamine dosages, a stabilization in metabolic disorders, and less-than-expected loss of extremities. Therapy proved to be safe and well tolerated. From this first experience, extracorporeal cytokine removal seems to be a valid and safe therapy in the management of meningococcal and pneumococcal diseases and may contribute to the patient stabilization and prevention of severe sequelae. Further studies are required to confirm efficacy in a larger context.

## 1. Introduction

Bacterial meningitis and septicemia are invasive bacterial diseases affecting, respectively, the central nervous system (CNS) and the blood, representing a significant cause of morbidity and mortality worldwide [1, 2] with rising risk of poor outcome in case of combination of these diseases. Reported in-hospital mortality range is between 24% [3] and 41% [4] and the most frequent causes of death are systemic complications in the elderly (>50 years of age) and cerebral complications in the younger patients (<2 years of age) [5].

*Neisseria meningitidis* (Gram-negative bacteria) and* Streptococcus pneumoniae* (Gram-positive bacteria) are the leading causative pathogens of invasive bacterial disease, with the latter being responsible for two-thirds of the reported cases in Western Europe and the US [5].

Once these bacteria have entered the bloodstream through the nasopharynx mucosa, they may survive and proliferate thanks to their polysaccharide capsule, provoking an immune and inflammatory reaction, which may rapidly lead to fulminant septic shock [6]. Then these bacteria may also invade the blood-brain barrier, causing bacterial meningitis and provoking an immanent inflammatory reaction in the CNS. This inflammation contributes to neuronal injury and involves the subarachnoid space, the meninges, and the brain parenchymal vessels. A hallmark of the inflammatory response associated with the infection is the excessive production of cytokines, chemokines, and other inflammatory mediators [6–9] with sometimes fulminant, devastating courses. This can lead to an uncontrolled, overwhelming reaction of the body's host response against itself leading to cerebral edema, infarction, hydrocephalus, and septic shock with multiple organ failure [10, 11].

Despite advances in critical care and early administration of antibiotics and corticosteroids, invasive bacterial diseases still represent a major challenge to physicians. Bacterial meningitis, if treated successfully, presents a high burden of sequelae so that approximately half of the survivors exhibit neurological sequelae [11]. Septicemia may be a dramatic fulminant consequence of bacterial meningitis or may be present on its own, increasing the high mortality rate of these patients [6–11].

A common dramatic feature is the occurrence of disseminated intravascular coagulation (DIC) [12], characterized by extensive activation of the coagulation system, amplified by inhibition of anticoagulant pathways, and often associated with the rapid onset of hypotension, acute adrenal hemorrhage, and multiorgan failure, leading to poor outcome [11, 12].

Despite the necessity of early recognition of symptoms and the prompt administration of antibiotics treatment, new adjunctive therapies are on their way. Inhibition of leukocyte recruitment or pattern recognition receptors, adjuvant erythropoietin/corticosteroid/complex vitamin B treatments, radical scavenging, and therapeutic hypothermia, have been described extensively elsewhere [8–10]. Since pneumococcal and meningococcal PAMPs act proinflammatorily provoking the generation of proinflammatory cytokines and chemokines, inhibition of these would seem a reasonable approach. Previous attempts to fight sepsis or the systemic inflammatory response syndrome (SIRS) by removing a single, specific cytokine have not been able to demonstrate an improvement in outcome. This brings up the question as to whether a broad, rather than specific, approach to remove the multitude of cytokines and other factors at fault in the disease may be more promising. In this context, extracorporeal blood purification therapies with the potential ability to alter the host inflammatory response through broad-spectrum, nonselective removal of inflammatory mediators have come into focus. A new hemoadsorption device, Cytosorb (Cytosorbents Corp, USA), intended as adjunctive treatment for patients with elevated cytokine levels in the setting of SIRS, sepsis, and septic shock has shown encouraging results in the evolution of these critical patients [13–18], but clinical cases are not available for meningococcal or pneumococcal infections.

Therefore, the aim of this case series is to report our clinical experience about the use of hemoadsorption for cytokine removal in that kind of patients.

## 2. Clinical Cases

We herein present five patients with pneumococcal or meningococcal sepsis early treated with Cytosorb for cytokine removal as adjunctive therapy.

Patient characteristics, treatments, clinical parameters, and patient outcome are presented in Table 1.

All the Cytosorb treatments were performed continuously until a hemodynamic, metabolic, and inflammatory improvement was observed, in combination with continuous renal replacement therapy (CRRT). Blood flows were set among 100 and 180 ml/min, according to the hemodynamic response of the patient, while dialysis doses were in the range of 30 and 35 ml/kg/h. A citrate-based protocol was used in all the patients.

### 2.1. Case 1

Case 1 is a 40-year-old male, presenting with general malaise, arthralgia in his extremities, facial cyanosis, and fever (39°C). In the medical history, a post-traumatic splenectomy was reported. Empirical antibiotic therapy started with ceftriaxone (2 g every 12h) and vancomycin (500 mg every 6h). After several bacteriological analysis, secondary sepsis caused by meningitis from* Streptococcus pneumoniae* was diagnosed. The patient was transferred to the intensive care unit (ICU) where his clinical status rapidly deteriorated, showing a severe hemodynamic instability with need for vasopressor support (norepinephrine 0.4 *μ*g/kg/min), persistent hypotension, and a pronounced inflammatory state with C-reactive protein (CRP) at 176.3 mg/l and procalcitonin (PCT) at 485.88 ng/ml. The patient also exhibited metabolic abnormalities, with lactate levels of 7.8 mmol/l, and severe coagulative disorders.

In face of a persistent anuria, continuous renal replacement therapy (CRRT), hemodiafiltration mode (CVVHDF, Prismaflex, M150, Baxter, USA) was started, together with Cytosorb cartridge, for a total of 68 hours (start of treatment <24h after ICU admission).

**Figure 1:**
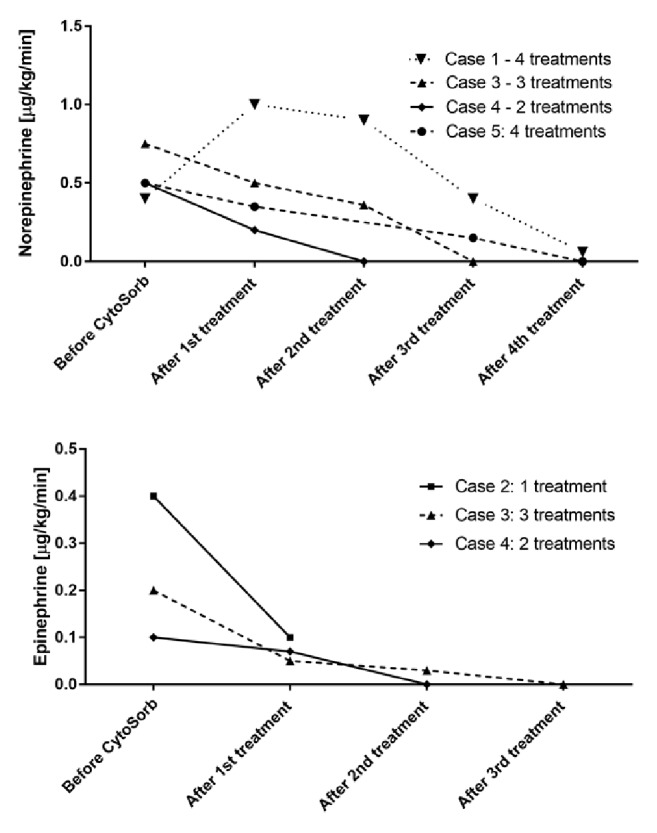
Course of norepinephrine and epinephrine demand during Cytosorb treatment in all treated patients. Values are shown at the beginning of the treatment and after every treatment until the end.

**Figure 2:**
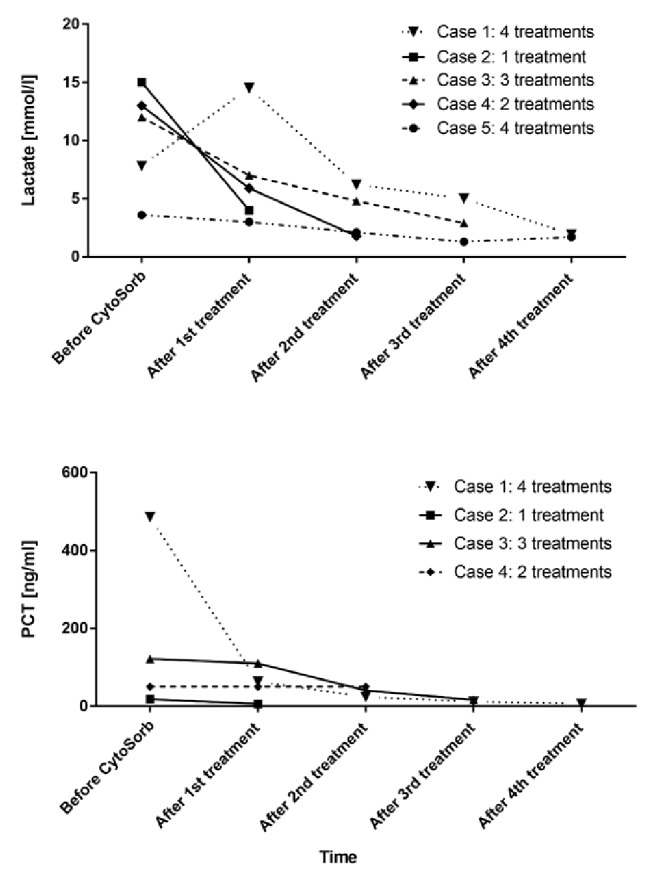
Course of lactate and PCT during Cytosorb treatment in all treated patients. PCT is not presented for Case 5 because it has not been measured with routine. Values are shown at the beginning of the treatment and after every treatment until the end.

A general improvement was obtained already during the first treatment. PCT could be reduced to 6 ng/ml at the end of the treatments. Similarly, CRP also dropped to 136.41 mg/l during the course of the treatments. On the metabolic level, the patient showed a marked improvement in lactate acidosis, with lactate levels decreasing to 1.9 mmol/dl. From a hemodynamic point of view, a MAP stabilization could be achieved paralleled with a reduction in the need for norepinephrine. The patient could be stopped from CRRT after 12 days with a recovery of diuresis. The course for norepinephrine demand during Cytosorb treatment is presented in Figure 1, whereas the course of lactate and PCT is shown in Figure 2.

The patient was still hospitalized after 2 months, however in an alert, collaborative, tracheotomised, spontaneously breathing state, waiting for transfer to a rehabilitation facility.

### 2.2. Case 2

Case 2 is a 66-year-old female, presenting with general illness, fever (40°C), and hypotension. Prior medical history included beta-blocker therapy for tachyarrhythmia, appendectomy, colon cancer, and splenectomy for colonic metastases. On admission, she exhibited skin ischemic lesions with rapid deterioration to whole body cyanosis and metabolic and lactic acidosis. Empirical antibiotic therapy started with ceftriaxone (2 g every 12h) and levofloxacin (500 mg every 12h). After 24h, blood cultures confirmed the diagnosis of sepsis from* Streptococcus pneumoniae* paralleled with severe purpura fulminans. Empirical antibiotic therapy continued for 11 and 7 days, respectively, with adjustments due to renal function changes. In the further course, she became oligoanuric, severely hypotonic with hemodynamic instability (epinephrine 0.4 *μ*g/kg/min), and thrombocytopenic, presenting severe bleeding.

A CVVHDF treatment (Multifiltrate, AV1000, Fresenius Medical Care, Germany) was started in combination with one 24-hour session of Cytosorb hemoadsorption immediately after the ICU admission.

This combined treatment resulted in a clear and progressive hemodynamic stabilization accompanied by a marked reduction of epinephrine to 0.1 *μ*g/kg/min (Figure 1) and a reduction of CRP, from 17.5 mg/dl to 13 mg/dl. Initially diuresis started to work again; however this went back to anuria in the first 12 hours (probably due to sepsis-associated tubular necrosis) and fully recovered later on day 10. Lactacidemia, elevated at the admission (15 mmol/l), decreased until 4 in the first 24h. At the same time, PCT decreased from 18.35 ng/ml to 2 ng/ml after 6 days (Figure 2).

Subsequent to this first acute event, the patient showed a recrudescence of the septic state. Blood cultures were positive for* Enterobacter aerogenes*, which colonise the body through the injured intestine wall, resulting from purpura fulminans. Because of her severe injuries at the extremities, the patient needed amputations and 2 months later, the reoccurrence of the second septic episode caused the death of the patient.

### 2.3. Case 3

Case 3 is a 47-year-old male showing fever (38°C), asthenia, and lumbar pain with signs of diffuse petechiae. In the further course, he became severely hypovolemic and tachycardic, showing also metabolic acidosis (lactate 12 mmol/l). Blood cultures were taken and empirical antibiotic therapy was started with meropenem (2 g every 8h) and amikacin (1 g every 24h). Diagnoses confirmed by blood cultures were septic shock secondary to* Neisseria meningitidis* infection (Serotype C) and antibiotic therapy was replaced with ceftriaxone (2 g every 12h). After transfer to the ICU, norepinephrine infusion was started at 0.2 *μ*g/kg/min and rapidly increased to 0.5 *μ*g/kg/min, in combination with epinephrine infusion at 0.2 *μ*g/kg/min. Inflammation was controlled with PCT, which was extremely elevated, 121.7 ng/ml. In the following hours, a worsening of general conditions of the patient was reported, with extension of petechiae and severe bleeding from the insertion points of the catheters and severe hypotension with the increase of norepinephrine up to 0.75 *μ*g/kg/min. In face of a persistent condition of oligoanuria, it was necessary starting a hemofiltration treatment (CVVH, Prismaflex, M150, Baxter, USA) and Cytosorb was additionally installed into the CVVH circuit the next day (start of treatment: 15h after ICU admission).

Within the course of 72-hour Cytosorb treatments, the patient witnessed a hemodynamic improvement with norepinephrine and epinephrine being tapered off after the third session (Figure 1) as well as a stabilization of lactate values and inflammation, with PCT decreased until 16.52 ng/ml (Figure 2).

The patient also showed an attenuation of disseminated intravascular coagulopathy, a demarcation of skin necrosis areas, and a recovery of vital functions. The patient could be extubated 10 days and weaned from CVVH 12 days after the ICU admission. He was transferred to rehabilitation medicine after 23 days from the recovery, waiting for plastic surgeon consultant for the amputation of some phalanges.

### 2.4. Case 4

Case 4 is a young male, presenting with fever (40°C), vomiting, pain in the lower limbs, and petechiae in extension with suspected disseminated intravascular coagulopathy and septic shock. He exhibited hemodynamic instability and metabolic acidosis (lactate 13 mmol/l). The patient was immediately transferred to ICU and pharmacological therapy with dexamethasone (10 mg every 6h) and immunoglobulins were immediately started. Diagnosis was septic shock secondary to* Neisseria meningitidis* infection (serotype C), confirmed by labs, and maxillary sinusitis was set as the probable cause of infection. Antibiotic therapy was then modified to ceftriaxone (2 g every 12h). The patient developed acute kidney injury and CRRT, hemodialysis mode, was started (CVVHD, Multifiltrate, AV1000S, Fresenius Medical Care, Germany).

Hemodynamic instability was observed with a median arterial pressure of 75 mmHg, necessitating administration of epinephrine at 0.1 *μ*g/kg/min and norepinephrine at 0.5 *μ*g/kg/min. In the following hours diuresis partially recovered; however the general critical condition did not improve and therefore Cytosorb was performed for a total of 32 hours in combination with CRRT (start of treatment: 11h after ICU admission). Three hours after start of Cytosorb, norepinephrine could be reduced to 0.2 *μ*g/kg/min and epinephrine to 0.07 *μ*g/kg/min. After the second treatment, norepinephrine and epinephrine infusion were stopped (Figure 1) and there was no more need for renal support. During the hemoadsorption treatment, we further noticed a clear stabilization of lactic acidosis from initially 13 to 1.78 mmol/l and a total recovery of diuresis. The course of lactate and PCT is shown in Figure 2.

The patient could be extubated 1 day after the cessation of Cytosorb treatment and transferred to paediatrics two days later in good medical condition without severe consequences or amputation need.

### 2.5. Case 5

Case 5 is a 36-year-old female, showing a persistent fever, severe polymyalgia, and diffuse petechiae in expansion to the limbs and arms. Antibiotic therapy was started with levofloxacin (750 mg every 24h) and ceftriaxone (2 g every 12h). The patient was immediately transferred to ICU with a suspected diagnosis of sepsis secondary to meningitis. The patient presented hypotension, requiring the administration of norepinephrine at 0.5 *μ*g/kg/min and dobutamine in the next day. Lab tests confirmed the diagnosis of bacterial meningitis infection caused by* Neisseria meningitidis* (Serotype C) and underlined a condition of thrombocytopenia, acute renal failure, and inflammation, initially monitored with CRP (17.5 mg/l). Lactate level was at 3.6 mmol/l. Immediately after the ICU admission, a CVVHDF treatment (Prismaflex, Oxiris, Baxter, USA) was started in combination with Cytosorb (start of treatment: 8h after ICU admission).

**Figure 3:**
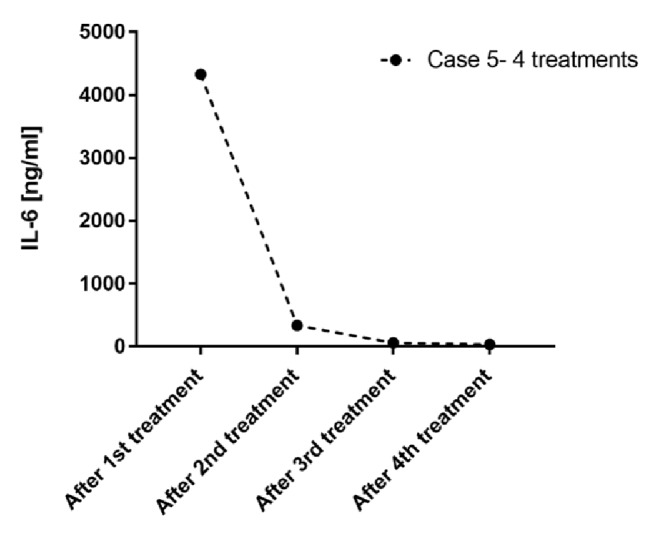
Course of IL-6 during Cytosorb treatment in Case 5. Values are shown after the first Cytosorb treatment, and then after every 24h until the end.

Within the course of hemoadsorption treatments, performed for a total of 96 hours, a hemodynamic stabilization was observed, associated with a reduction in need of inotrope drugs. Indeed, dobutamine could be stopped after 48h and norepinephrine after 78h. The course of norepinephrine is shown in Figure 1. The patient showed a stabilization lactate level, reduced at 1.7 mmol/l, as presented in Figure 2. Moreover, the inflammatory status was also monitored during Cytosorb treatment dosing IL-6 levels (Figure 3), directly adsorbed by the sorbent. After 12h of hemoperfusion, IL-6 values were 4.326 ng/ml and decreased dramatically during the course of treatments until 60.1 ng/ml after 60h and 35 ng/ml after 72h. Petechiae and skin lesions were stable already after the first day of treatment. A complete recovery of renal function and diuresis was observed after 9 days from the admission and the patient could be transferred to medicine after 7 days. The patient was followed by dermatologists and vascular surgeons for the outcome of the septic embolism complication, evaluating the possible amputation of one toe.

## 3. Discussion

Morbidity and mortality for bacterial meningitis and sepsis remain high [1–4]. The risk factors for a poor outcome, besides other medical conditions, include systemic compromise and a low level of consciousness. Importantly, outcome largely depends on rapid initiation of an effective empiric treatment [5–9]. Despite the necessity of early recognition of symptoms and the prompt administration of antibiotics, new adjunctive extracorporeal therapies [8, 9] focused on the control of the inflammatory response caused by pneumococcal and meningococcal bacteria would seem a reasonable approach [13–18].

These case reports appear to be among the first published applications of Cytosorb as an adjunctive treatment in the management of meningococcal and pneumococcal sepsis. The combined treatment of CRRT and Cytosorb was well tolerated in all patients.

The main results to be drawn from our experiences are predominantly a progressive and clear stabilization in hemodynamics along with a rapid and marked reduction of catecholamine dosages (Figure 1). Moreover, we consistently recorded a stabilization in metabolic disorders as seen by a decrease in lactate levels (Figure 2). Except for one patient, dying after 2 months from a secondary septic episode, all patients survived. All the patients showed also a stabilization within normal range of coagulation parameters, showing an attenuation of the embolic septic status. Importantly, despite commonly occurring coagulation disorders leading to amputations, we noticed only minor loss of extremities in three patients (some phalanges and one toe) or even no need for an amputation in one patient.

Two of our patients had undergone splenectomy, one with a post-traumatic background and the other by elective surgery due to colonic metastases. While the first patient survived, the latter died. Importantly, hyposplenic patients are at high risk for overwhelming sepsis caused by pneumococcal bacteria [3].

The patients described herein have all been treated early between 8 and 24 hours after initial diagnosis. A prospective study performed on two ICUs over a period of 6 years in patients with acute community-acquired bacterial meningitis could show that the overall severity of the disease within 24 hours of admission may be the major indicator of adverse in-hospital clinical outcome [10].

A recently published case series underlined the more pronounced effects of Cytosorb treatment in septic shock patients when therapy was started within 24 hours of sepsis diagnosis. On the other hand, a delay in the start of therapy was associated with a poor response in terms of reduction of catecholamine demand and survival [13].

As underlined in literature [13–18], we performed each hemoadsorption treatment for 24 hours, except in two patients (cases 1 and 4) in which we changed the entire extracorporeal circuit after approximately 12 hours due to the severe coagulative disorders of the patients at the beginning. The continuation of the treatment was decided in function of the patient clinical improvement. In particular, the therapy was continued until catecholamine demand was stopped (cases 3, 4, and 5) or drastically decreased from the beginning of the treatment (cases 1 and 2) and an important reduction in lactate levels was observed (in all cases, lactate levels normalized around the reference limit value).

Overall, an improvement in hemodynamics seems to be one of the consistent key clinical benefits seen with this therapy, also confirmed in preclinical studies and published evidences [13–18].

Regarding the inflammatory response in these patients, pneumococcal and meningococcal compounds are extremely aggressive because of their polysaccharide capsule, letting them to escape from phagocytosis in blood and once recognized by specified receptors, they trigger an expression of inflammatory cytokines, chemokines, and reactive oxygen species [6]. Their excessive and uncontrolled action might have harmful effects and once they become systemic, sepsis with multiple organ failure might occur. Cutting off these high peak-plasma levels of several inflammatory mediators would be desirable [6–9].

Although cytokine levels were not recorded, except in one patient (Figure 3) which showed an impressive reduction of IL-6, as these types of measurements are not routine in all institutions, cytokine reduction may be at the background of the hemodynamic and metabolic improvements in all patients [13–18].

The inflammatory response modulation might have a benefit effect also on the coagulation status because it has been found that cytokines influence both procoagulant and anticoagulant pathways [6]. Therefore, their control might contribute to the recovery of homeostasis, in addition to the action on microcirculation, leading to an improvement in blood perfusion through blocked vessels [13–18].

All patients were empirically treated with broad-spectrum antibiotics and steroids without change in dosage.

## 4. Conclusion

To our knowledge, this is the first report on the successful use of hemoadsorption for cytokine removal therapy in a set of patients with meningococcal and pneumococcal sepsis. Effects associated with its application included a rapid and clear stabilization in hemodynamics along with a reduction in catecholamine dosages, a decrease in lactate as well as a less-than-expected loss of extremities. Therapy proved to be safe and well tolerated. After these promising initial data, a solid proof of efficiency is needed for the implementation in a larger context.

## Figures and Tables

**Table 1:** Patient characteristics, treatments, clinical parameters, and patient outcome. M: male, F: female, Cat-free: catecholamine-free, CRRT: continuous renal replacement therapy, SOFA: Sequential Organ Failure Assessment, and PELOD: Paediatric Logistic Organ Dysfunction (*∗*only case 4).

***Case number***	*1*	*2*	*3*	*4∗*	*5*

***Sex***	M	F	M	M	F

***Age***	40	66	40	14	36

***Diagnosis***	Pneumococcal Sepsis	Pneumococcal Sepsis	Meningococcal Sepsis (Serotype C)	Meningococcal Sepsis (Serotype C)	Meningococcal Sepsis (Serotype C)

***Antibiotics***	Ceftriaxone/ Vancomycin	Ceftriaxone/ Levofloxacin	Meropenem/ Amikacin/ Ceftriaxone	Dexamethasone/ Ceftriaxone	Levofloxacin / Ceftriaxone

***Cytosorb treatments (n)***	4	1	3	2	4

***Cytosorb treatment time (h)***	68	24	72	32	96

***Delay (h)***	<24	<15	15	11	8

***SOFA Score/*** **∗** ***PELOD Score Admission***	13	17	18	22	12

***SOFA Score/*** **∗** ***PELOD Score Post treatment***	10	20	16	13	8

***Cat-free (days)***	6	3	5	3	4

***CRRT (days)***	12	10	12	4	5

***Ventilation (days)***	20	53	10	4	5

***ICU stay (days)***	49	53	17	6	7

***Amputation need***	Yes/Phalange	Yes/Limbs	Yes/Phalanges	No	Yes/Toe

***ICU mortality***	No	Yes	No	No	No

***28-day survival***	Yes	Yes	Yes	Yes	Yes

***Hospital mortality***	No	Yes	No	No	No

## Data Availability

All data analysed during this study are included in this published article.

## References

[1] van de Beek D., Brouwer M. C., Thwaites G. E., Tunkel A. R. (2012). Advances in treatment of bacterial meningitis. *The Lancet*.

[2] van de Beek D., de Gans J., Tunkel A. R., Wijdicks E. F. M. (2006). Community-acquired bacterial meningitis in adults. *The New England Journal of Medicine*.

[3] Kastenbauer S., Pfister H.-W. (2003). Pneumococcal meningitis in adults: Spectrum of complications and prognostic factors in a series of 87 cases. *Brain*.

[4] Muralidharan R., Mateen F. J., Rabinstein A. A. (2014). Outcome of fulminant bacterial meningitis in adult patients. *European Journal of Neurology*.

[5] Brouwer M. C., Tunkel A. R., van de Beek D. (2010). Epidemiology, diagnosis, and antimicrobial treatment of acute bacterial meningitis. *Clinical Microbiology Reviews*.

[6] Pathan N., Faust S. N., Levin M. (2003). Pathophysiology of meningococcal meningitis and septicaemia. *Archives of Disease in Childhood*.

[7] van de Beek D., de Gans J., Spanjaard L., Weisfelt M., Reitsma J. B., Vermeulen M. (2004). Clinical features and prognostic factors in adults with bacterial meningitis. *The New England Journal of Medicine*.

[8] Nau R., Djukic M., Spreer A., Eiffert H. (2013). Bacterial meningitis: New therapeutic approaches. *Expert Review of Anti-infective Therapy*.

[9] Barichello T., Collodel A., Generoso J. S. (2015). Targets for adjunctive therapy in pneumococcal meningitis. *Journal of Neuroimmunology*.

[10] Flores-Cordero J. M., Amaya-Villar R., Rincón-Ferrari M. D. (2003). Acute community-acquired bacterial meningitis in adults admitted to the intensive care unit: Clinical manifestations, management and prognostic factors. *Intensive Care Medicine*.

[11] Hoogman M., van de Beek D., Weisfelt M., de Gans J., Schmand B. (2007). Cognitive outcome in adults after bacterial meningitis. *Journal of Neurology, Neurosurgery & Psychiatry*.

[12] Thachil J. (2016). Disseminated Intravascular Coagulation: A Practical Approach. *Anesthesiology*.

[13] Kogelmann K., Jarczak D., Scheller M., Drüner M. (2017). Hemoadsorption by CytoSorb in septic patients: A case series. *Critical Care*.

[14] Träger K., Skrabal C., Fischer G. (2017). Hemoadsorption treatment of patients with acute infective endocarditis during surgery with cardiopulmonary bypass—A case series. *The International Journal of Artificial Organs*.

[15] Träger K., Fritzler D., Fischer G. (2016). Treatment of post-cardiopulmonary bypass SIRS by hemoadsorption: A case series. *The International Journal of Artificial Organs*.

[16] Peng Z.-Y., Carter M. J., Kellum J. A. (2008). Effects of hemoadsorption on cytokine removal and short-term survival in septic rats. *Critical Care Medicine*.

[17] Hinz B., Jauch O., Noky T., Friesecke S., Abel P., Kaiser R. (2015). CytoSorb, a novel therapeutic approach for patients with septic shock: a case report. *The International Journal of Artificial Organs*.

[18] Friesecke S., Stecher S.-S., Gross S., Felix S. B., Nierhaus A. (2017). Extracorporeal cytokine elimination as rescue therapy in refractory septic shock: a prospective single-center study. *The International Journal of Artificial Organs*.

